# Throughput of an IEEE 802.11 Wireless Network in the Presence of Wireless Audio Transmission: A Laboratory Analysis

**DOI:** 10.3390/s21082620

**Published:** 2021-04-08

**Authors:** Ivan Forenbacher, Siniša Husnjak, Ivan Jovović, Mislav Bobić

**Affiliations:** Department of Information and Communication Traffic, Faculty of Transport and Traffic Sciences, University of Zagreb, Vukelićeva 4, 10 000 Zagreb, Croatia; sinisa.husnjak@fpz.unizg.hr (S.H.); ivan.jovovic@fpz.unizg.hr (I.J.); mislav_bobic@hotmail.com (M.B.)

**Keywords:** IEEE 802.11, Industry 4.0, communication requirements, non-Wi-Fi interference, wireless network, throughput measurement, practical implications

## Abstract

Wireless networks, including IEEE 802.11-based or Wi-Fi networks, are inexpensive and easy to install and therefore serve as useful connectivity alternatives in areas lacking wired-network infrastructure. However, IEEE 802.11 networks may not always provide the seamless connectivity and minimal throughput required for Industry 4.0 communications because of their susceptibility to interference from other devices operating in the unlicensed “Industrial, Scientific, and Medical” frequency band. Here we analyzed how a wireless audio transmitter operating on this band influences the throughput of an IEEE 802.11 b/g/n network under laboratory conditions. Wireless audio transmission reduced mean throughput by 85%, rendering the IEEE 802.11 b/g/n network nearly unusable. Our analysis suggests that in order for IEEE 802.11 wireless networks to support Industrial 4.0 applications, attention should be paid to the physical layer as well as the data or upper layers, and critical services should not transmit on the 2.4 GHz band. These findings may contribute to understanding and managing IEEE 802.11 wireless networks in various Industry 4.0 contexts.

## 1. Introduction

Industry 4.0 refers to the next evolutionary step of industrial development that enables interconnection, automatization, and digital management of industrial ecosystems [1]. Industry 4.0 has become the focus of many researchers, manufacturers, and governments as a way to make factories *smart* by optimizing energy consumption and resource use [1,2].

Industry 4.0 may benefit from wireless networks, which have proliferated in many environments as a low-cost and high-performing “workaround” to physical wired networks in environments where such infrastructure is prohibitively expensive or challenging to install and maintain [1,3]. Wireless networks offer numerous advantages over wired networks, such as mobility and scalability, low installation costs, and independence from physical damage or deterioration of cables [4]. A wireless alternative that is particularly promising for Industry 4.0 is IEEE 802.11-based wireless networks [3,4], which are similar to the Open System Interconnection (OSI) model and whose protocol architecture resembles that of Ethernet-based wired networks [4]. This ensures extensive, smooth interoperability between Ethernet and IEEE 802.11-based networks [4].

On the other hand, IEEE 802.11 networks are vulnerable to interference or even deliberate jamming [3,4], which can lead to network failure [2]. This may prevent IEEE 802.11 networks from fulfilling the Industry 4.0 requirement for seamless connection between industrial components and devices [3,5]. Since these networks operate in unlicensed wireless bands such as the “Industrial, Scientific, and Medical” (ISM) band, they may receive interference from other devices operating on those bands, such as unmanned aerial vehicles (drones) flying over smart warehouses, audio/video transmitters, cordless phones, or Bluetooth handsets operating near industrial devices. Such interference can occur at any time and degrade the quality of network service, such as by reducing throughput or even interrupting wireless communication entirely [2].

Previous studies have focused on the signal coverage of IEEE 802.11 wireless networks and neglected potential interference from non-IEEE 802.11 devices and the impact on network performance [6,7]. Therefore, the present study measured the effect of wireless audio transmission on an IEEE 802.11 network in a laboratory environment. Our results may help network and IT professionals as well as end-users manage and troubleshoot non-IEEE 802.11 interference in IEEE 802.11 networks.

## 2. Literature Review

### 2.1. Applications and Challenges of IEEE 802.11 Wireless Networks in Industry 4.0

The general Industry 4.0 framework consists of two critical layers: (1) devices and (2) networks. The first layer comprises mobile devices, sensors, machines, automated guided vehicles, as well as people and other data-collecting entities that communicate with one another [2]. This communication is made possible by the second layer, which comprises wired and wireless communication technologies that allow real-time data transfer among devices in the first layer [2].

Regarding the second layer, Industry 4.0 already complements wired networks by making use of various wireless communication technologies (Table 1) which have to respond to various industrial requirements towards throughput, seamless connectivity, dynamic topologies, signal interference and path loss, real-time performance, and reliability.

IEEE 802.11 networks are able to fulfill those requirements, as well as being widely available and easy and inexpensive to install, which makes them important wireless communication technology in Industry 4.0 [2,4,12]. For example, IEEE 802.11 networks have been used in various Industry 4.0 contexts, such as production planning, monitoring, data collection, navigation of moving devices, and transfer of multimedia content [3,13]. Studies have examined IEEE 802.11 networks for monitoring steam flood and water flood pipelines in oil fields [14], monitoring a water pump control system [15], networking and managing automated guided vehicles in a warehouse [16], broadcasting multimedia content in real time [1,13,17], and exchanging multimedia data between industrial PCs and monitoring devices in real time [13].

Thus, IEEE 802.11 networks can connect many devices in a heterogeneous industrial environment to support diverse applications, but they must fulfill certain minimum requirements to support the high demands of Industry 4.0, such as high throughput, including for a rapidly expanding range of multimedia applications [2,3,5], as well as dynamic topology that ensures uninterrupted communication in the presence of movement and large fluctuations in throughput [3]. Working against these performance challenges is interference from other wireless devices that also operate in the ISM band [2]. Data from these devices can “collide” on the network with data transmitted by desired devices, increasing the bit error rate and reducing throughput [2,18,19,20,21].

In order to understand, predict, and manage the effects of external wireless devices on IEEE 802.11 networks, research is needed about their effects on throughput, the primary measure of a network’s quality of service [3,6,7]. This requires analyzing the physical (PHY) layer next to the data (MAC) layer of OSI model [22].

### 2.2. Measuring Effects of Non-IEEE 802.11 Interference on the Performance of IEEE 802.11 Net-Works

Several studies have examined the effects of interference from various non-IEEE 802.11 devices on the performance of IEEE 802.11 networks. For example, one study found that microwave ovens decreased throughput by 28–49%, depending on proximity to the network (1–3 m), whereas Bluetooth loudspeakers reduced it by 5–9% [22]. Another study even found that microwave ovens could stop network throughput entirely [7]. Interference from microwave ovens can be mitigated using a “cognitive radio” technique [23]. In other work, Bluetooth devices were shown to substantially increase the bit error rate of IEEE 802.11b networks [24], while Zigbee (IEEE 802.15.4), a wireless communication protocol intended for smart homes and other personal area networks with relatively low throughput and energy consumption, reduced throughput by 20–40% [25]. One study found that, when sufficiently close to an IEEE 802.11 network, a Bluetooth handset reduced throughput by 26.5%, and a videophone reduced it by 7.5% [26]. On the other hand, another study [27] concluded that neither microwave ovens nor Bluetooth devices affect throughput of down- or uploads, but that they do weaken IEEE 802.11 signal strength as well as the wall between the client and access points.

Radiofrequency noise from a laptop computer can reduce throughput and other aspects of IEEE 802.11 network performance [28]. Analogue wireless video cameras and analogue cordless phones can reduce network throughput by 90–100% because they transmit continuously and therefore interfere with network traffic most of the time [7].

Despite these studies of various types of non-IEEE 802.11 interference, we are unaware of studies analyzing the effects of wireless audio transmitters on the throughput of IEEE 802.11 wireless networks. Wireless audio transmitters and wireless audio handsets may be used for warehouse sound systems or augmented reality purposes in Industry 4.0 contexts [29,30]. Therefore, the present study measured the effects of a Line6 Relay G30 wireless audio transmitter on throughput of an IEEE 802.11 network in a laboratory environment. We selected this transmitter model as a reasonable proxy for the range of wireless audio devices that can be found in Industry 4.0 environments.

## 3. Effects of Interference on the PHY and MAC Layers of an IEEE 802.11 Network

IEEE 802.11 networks operate at frequency bands of 2.4 and 5 GHz, which offer a maximum of 14 or 24 channels, respectively, though the number of usable channels depends on the region [31]. These frequency bands fall within the unlicensed ISM band, which is also used for non-IEEE 802.11 communications. IEEE 802.11 devices exchange frames on the MAC layer. Three types of frames are used: (1) management frames, which are used for joining and leaving the IEEE 802.11 network; (2) control frames, which are used to acknowledge received frames; and (3) data frames, which contain the data. Frames are broadcast wirelessly using half-duplex communication.

**Figure 1:**
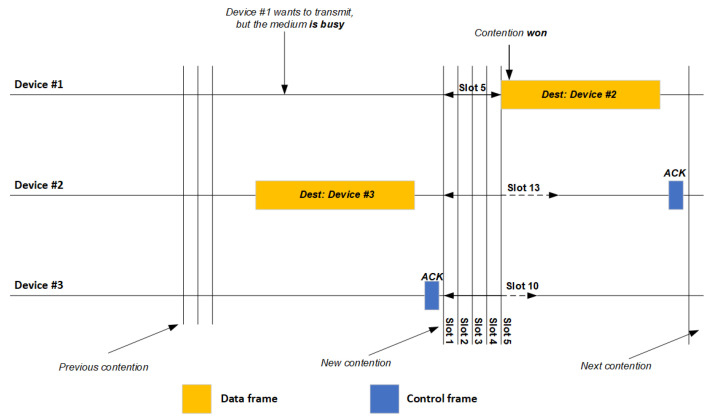
Illustration of Carrier Sense Multiple Access with Collision Avoidance (CSMA/CA) involving three devices on one channel of an IEEE 802.11 network without interference. ACK, Acknowledgment Control Frame; Dest, Destination.

To minimize the probability of collision on the network during such communication, IEEE 802.11-based devices check whether the channel is busy or idle before transmission. They perform this check using the Carrier Sense Multiple Access with Collision Avoidance (CSMA/CA) algorithm [31,32]. The device periodically samples the channel for the presence of radiofrequency energy, while a ‘carrier sense’ algorithm detects and encodes/decodes the IEEE 802.11 frames [32]. If the channel is idle, the first frame in the queue is transmitted. If the channel is busy, the device will wait for the current transmission to end and then “contention” starts: the device waits for a randomly determined time period or for a number of fixed-duration time slots (expressed in µs), then re-checks the channel to see whether it is now idle [33]. If so, the device sends the frame. The device with the shortest random time wins and sends its frame [33,34,35]. In this way, the IEEE 802.11 network uses a “stop-and-go” mechanism to provide all devices with equal probability of accessing the channel. In other words, the next frame in the queue is transmitted only if the current frame is acknowledged appropriately by the receiving device within the acknowledgment control frame (ACK) [33,34,35]. This entire process is illustrated in Figure 1.

**Figure 2:**
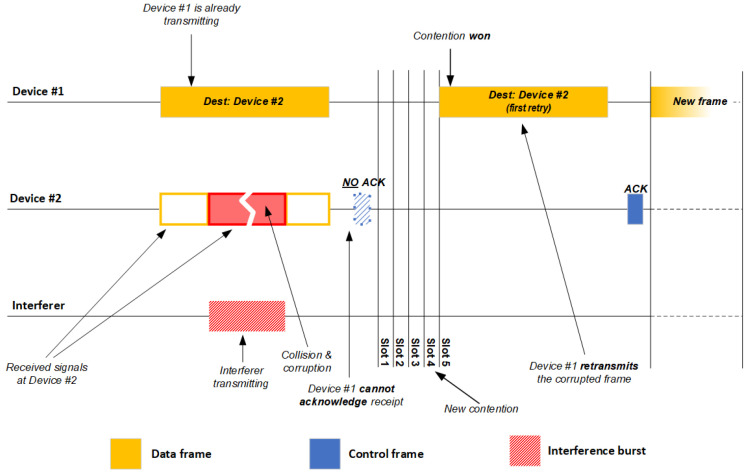
Illustration of CSMA/CA involving two devices and one interfering device on one channel of an IEEE 802.11 network in the presence of sporadic interference. ACK, Acknowledgment Control Frame; Dest, Destination.

**Figure 3:**
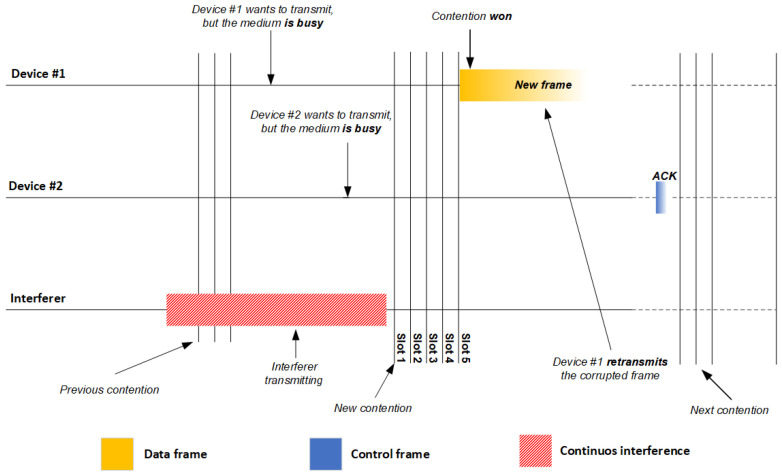
Illustration of CSMA/CA involving two devices and one interfering device on one channel of an IEEE 802.11 network in the presence of severe interference. ACK, Acknowledgment Control Frame.

This process can proceed quite differently in the presence of sporadic or continuous interference [4,35,36]. Such interference can be due to other IEEE 802.11 networks in the vicinity, or to Wi-Fi or non-Wi-Fi devices in the vicinity [22], such as cordless phones, Bluetooth handsets, audio and video transmitters, and microwave ovens. If the interferer transmits sporadically (Figure 2), an IEEE 802.11 device may receive signals both from the source IEEE 802.11 device and from the interferer. This may cause frame collision and corruption, which prevents the receiving device from acknowledging delivery of the frame (NO ACK) [34,35]. This necessitates retransmission of the corrupted frame as soon as the channel becomes idle again. Repeatedly unsuccessful delivery of data frames and retransmissions decrease network throughput. This problem is even worse if interference is severe and continuous (Figure 3). In this case, IEEE 802.11 devices consider the medium busy most of the time and do not transmit [34,35].

## 4. Equipment and Methods

### 4.1. Equipment

*IEEE 802.11 client*. An Apple iPhone SE (manufactured in 2016) was used as the IEEE 802.11 client. This model supports IEEE 802.11a/b/g/n/ac standards, as well as IEEE 802.11 tethering. It also supports HSPA, GSM, CDMA, EVDO and LTE. The device belongs to Category 4 according to the LTE-A standard.

**Figure 4:**
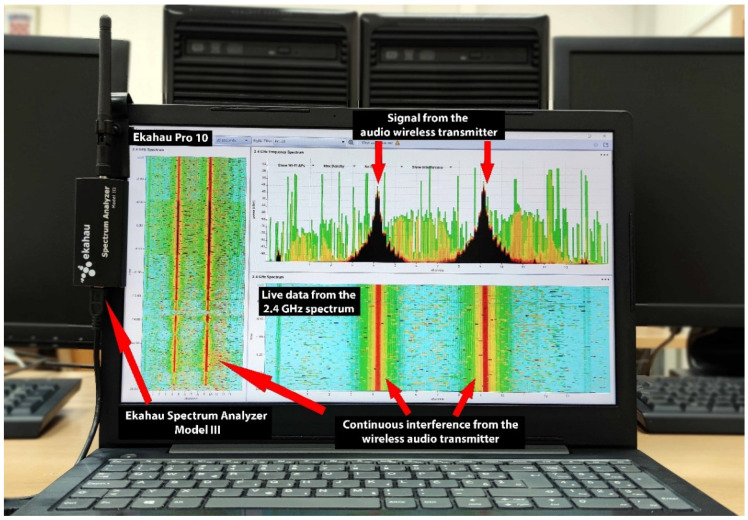
Spectrum analyzer comprising an Ekahau Spectrum Analyzer Model III and Ekahau Pro 10 software. The image shows data collected during a preliminary network run in the presence of interference.

*Non-IEEE 802.11 interferer*. A Line6 Relay G30 wireless audio transmitter was used. It was set up to operate in RF2 mode with two channels (2428 and 2453 MHz). The wireless audio transmitter created two spikes in the 2.4 GHz spectrum (Figure 4), which were present on IEEE 802.11 channels 3, 4, 5, 6, 8, 9, 10, and 11. Manufacturer specifications indicate a range up to 30 m, depending on the surroundings.

*IEEE 802.11 access point*. The D-Link DIR-615 access point was used (Table 2), which supports the IEEE 802.11 b, g and n standards. We opted for these standards because they are still widely used in various industry environments [2,3,4,5]. It has two fixed omni-directional antennas with a gain of 2 dBi. The access point was secured with WPA/WPA2 authentication to prevent any other unwanted clients to connect to our network.

We adopted the approach from [22] and opted for channel 6 in order to avoid potential interference from IEEE 802.11 networks near our laboratory set-up. Continuous monitoring using a spectrum analyzer detected only one additional IEEE 802.11 access point on channel 6, whose signal was weaker than −80 dBm–much weaker than the > −40 dBm signal of our network set-up. This monitoring confirmed minimal use of channel 6 during measurements.

*Spectrum analyzer*. A spectrum analyzer allowed us to examine the channel utilization and recognize various radiofrequency signal patterns in the PHY layer (Figure 4). An Ekahau Spectrum Analyzer Model III (Ekahau, Helsinki, Finland) was used to collect radiofrequency spectral data. This device has an external RP-SMA antenna, and it detects the range between −100 and −6.5 dBm at a resolution of 0.5 dBm. It supports both 2.4 and 5 GHz bands. The radiofrequency spectral data were represented in real time using Ekahau Pro 10 software, which allowed us to monitor the radiofrequency spectrum before and during measurements.

*Measurement of throughput*. The Speedcheck Internet Speed Test (https://apps.apple.com/us/app/speedcheck-internet-speed-test/id616145031, accessed on 26 March 2021) was used to measure the throughput of the wireless network. This test is ranked number 1 among applications for measuring network throughput on the Apple App Store. The application relies on an iOS-based client and a worldwide network of high-speed servers to measure throughput in three steps: (1) the client establishes multiple connections with the closest throughput server, then (2) the client application down- or uploads a certain amount of data, and finally (3) the time needed to complete the down- or upload is used to calculate throughput. We used an internet-based throughput test in order to emulate real-world industrial environments, where traffic needs to leave the local network. The inclusion of a much wider network ecosystem, as well as type of IEEE 802.11 client and access point used, may introduce variability, and prevent accurate determination of absolute down- and upload rates, but our focus was on *differences* between data transfer rates in the presence or absence of interference. We wanted to investigate whether interference from the wireless audio transmitter could be a dominant factor in degrading IEEE 802.11 network throughput.

### 4.2. Wireless Network Settings in the Laboratory Set-Up

**Figure 5:**
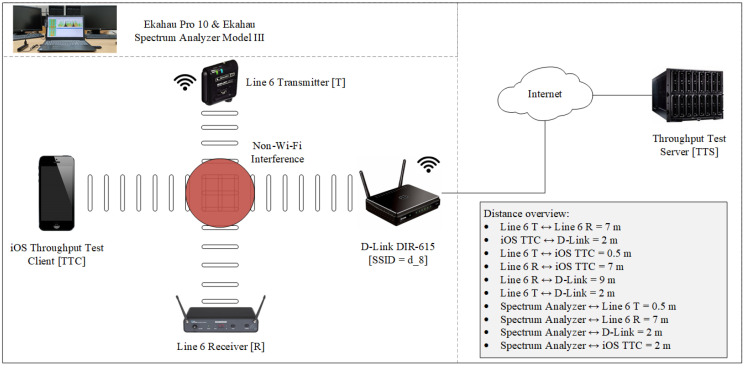
Network architecture settings for analyzing the effects of non-IEEE 802.11 interference on throughput of a wireless network under laboratory conditions.

We followed the approach from [22] and conducted our measurements under conditions as close as possible to those of an anechoic Faraday cage. A network architecture was set up in the Laboratory for Modeling and Optimizing Information and Communication Networks and Services at the Department of Information and Communication Traffic (Figure 5; Department’s official web site: https://www.fpz.unizg.hr/ikp/eng.php, accessed on 26 March 2021). The architecture consisted of an iOS mobile phone with a pre-installed client for measuring throughput, IEEE 802.11b/g/n access point, workstation with spectrum analyzer, Line6 Relay G30 wireless audio transmitter, and server for measuring throughput. The path for down- and uploading for measuring throughput was iOS Throughput Client → D-Link DIR-615 access point → Internet → Throughput Test Server.

### 4.3. Throughput Measurement and Data Analysis

**Figure 6:**
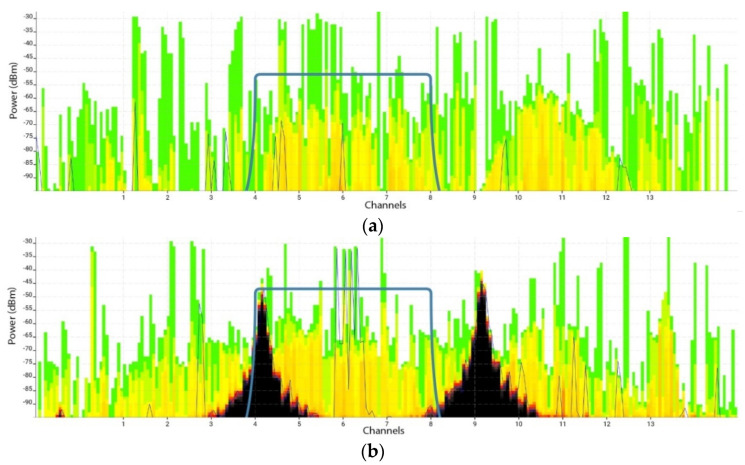
Time-averaged view of radiofrequency spectrum on the physical (PHY) layer on the 2.4 GHz band in the (**a**) no interference and (**b**) interference scenarios by averaging over 20 s. The blue line represents d_8 signal, while the two black peaks indicate signal from the sound transmitter. Darker color indicates greater channel use.

**Figure 7:**
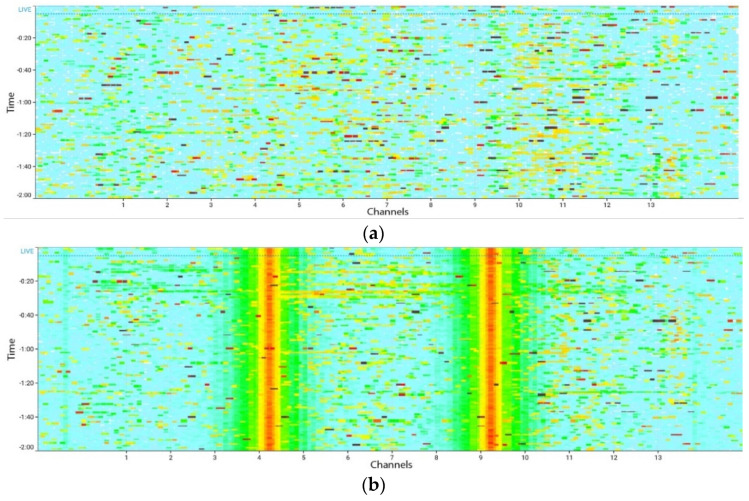
Radiofrequency spectrum activity over time on the PHY layer on the 2.4 GHz band in the (**a**) no interference and (**b**) interference scenarios. The two red vertical lines indicate continuous transmission from the sound transmitter. Darker color indicates greater signal strength.

Throughput was measured for the control scenario (no interference), when the Line6 Relay G30 wireless sound transmitter was turned off (Figure 6a and Figure 7a); and for the interference scenario, when the sound transmitter was turned on (Figure 6b and Figure 7b).

Throughput was measured in a total of 120 measurements taken in four steps: step 1 was 30 download measurements in the control scenario; step 2, 30 download measurements in the interference scenario; step 3, 30 upload measurements in the control scenario; and step 4, 30 upload measurements in the interference scenario. During all four steps, all devices in the network were kept at a constant distance from one another. All measurements were taken exclusively at periods when channel use was minimal in order to minimize interference from Wi-Fi and non-Wi-Fi sources. We confirmed minimal interference by continuously monitoring with a spectrum analyzer.

Measurements were analyzed in four steps. Step 1 involved obtaining mean values for down- and upload throughput values for each scenario. In step 2, the mean values for down- and upload were tested for normality in order to select Bartlett’s or Levene’s test for assessing homogeneity of variance in step 3. These steps were performed separately for data obtained in the control or interference scenarios. In step 4, the appropriate two-sample *t* test was selected and used to test whether mean throughput differed significantly between the two scenarios. The null hypothesis (H_null_) was defined as no significant difference between the two mean throughput rates.

## 5. Results

**Figure 8:**
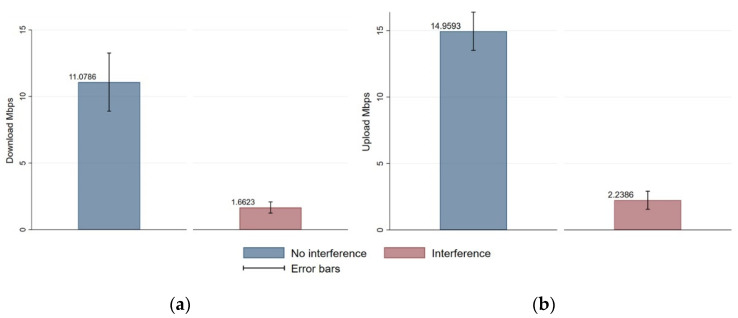
Mean throughput in the absence or presence of interference, based on download (**a**) and upload (**b**). Mean values are shown atop each bar.

Comparison of mean down- and upload rates prior to any significance testing indicated that interference reduced the mean download throughput from 11.09 ± 5.85 Mbps to 1.66 ± 1.12 Mbps (Figure 8). Similarly, interference reduced mean upload from 14.96 ± 3.85 Mbps to 2.24 ± 1.82 Mbps.

To evaluate the validity of our approach of using internet-based throughput measurements we performed additional measurements using the same setup from Figure 5 but this time we used a laptop (Lenovo V330, Lenovo Group Limited, Hong Kong, China) as the client device and a local desktop computer from the laboratory as the throughput server.

We followed the approach from [22] and performed 180 measurements of throughput for each scenario using *iPerf* application. *iPerf* is a tool used by network professionals for measuring maximum achievable data rates on IP networks. It was configured to use the default TCP window size of 64 KB. You can find more information on https://iperf.fr/ (accessed on 26 March 2021). Since the measurements were performed only on the local wireless link, it allowed us to eliminate any potential variability due to a wider network ecosystem and internet-based throughput test.

Measurements were taken during time window of 180 s in the absence and in the presence of interference. Interference from the wireless audio transmission reduced the mean throughput rates from 49.38 ± 1.55 Mbps to 6.51 ± 3.54 Mbps or 86%. This aligns to the previous drop of 85%. This suggests that focusing only on differences between data rates in the presence or absence of interference to determine whether the interference from wireless transmission could be a dominant factor in degrading IEEE 802.11b/g/n network throughput was optimal strategy and allowed us to confidently use our initial data and results to continue with further analysis.

**Figure 9:**
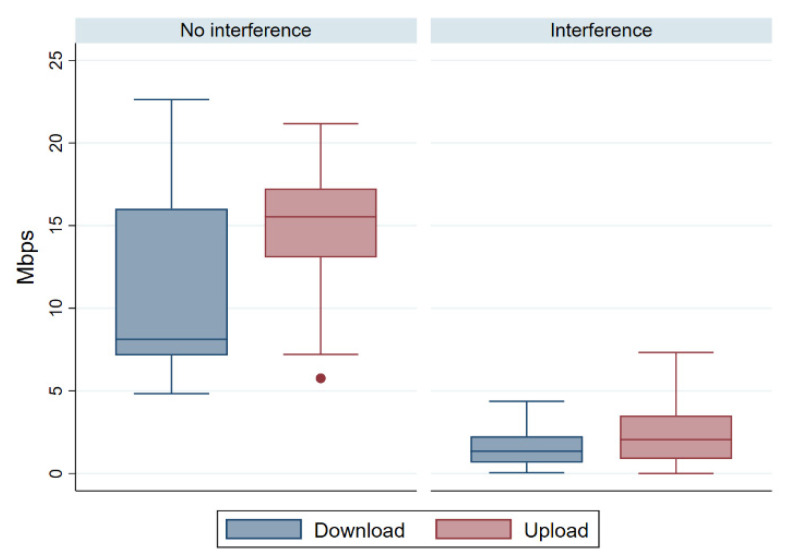
Tukey box plot of down- and upload throughput. The horizontal line drawn through the rectangles represents the median, while the upper and lower edges of the rectangles represent the interquartile range.

To examine whether these differences were significant, we first evaluated the normality of the data based on Tukey box plots (Figure 9) and tests for skewness or kurtosis (Table 3). Since the results indicated the possibility of skew, we opted for Levene’s test to assess whether variance was homogeneous, since this test is more robust to possible violations of normality [37]. This test indicated that variance was unequal for down- and upload measurements (*p* < 0.001; Table 4 and Table 5).

Finally, a *t* test based on unequal variances was performed to assess whether interference significantly reduced throughput (Table 6 and Table 7). In other words, we wanted to test if there is significant difference in down- and upload mean data rates between interference and no interference scenario. We opted for *t* test based on unequal variances since the results from the previous step and Levene’s test (Table 5 and Table 6) showed we can reject the null hypothesis that the variances are equal. The results indicated that, indeed, the reduction was statistically significant for both down- and upload.

## 6. Discussion

Reliable and seamless wireless communication is critical in industrial environments, so the effects of interference on IEEE 802.11 network performance should be understood in detail in order to allow the design of appropriate prevention and management measures [2]. In our laboratory set-up, interference from a wireless audio transmitter significantly reduced mean network throughput by 85%, based on measurements of down- and uploads.

The strong reduction in throughput observed here reflects that the network and interferer were operating on the same frequency band (2.4 GHz) and that the interferer was operating continuously. As a result, the IEEE 802.11 station detected non-IEEE 802.11 radiofrequency energy from the interferer on the PHY layer, so the station either backed off from accessing the channel or it transmitted data but at higher probability of collision or frame corruption. The throughput reduction observed here is comparable to that reported for analogue wireless cameras or analogue cordless phones, which also transmit continuously [7].

The reduction observed here is greater than the 49% reported for a microwave oven placed 1 m from the IEEE 802.11 access point and the 9% reported for Bluetooth speakers [18]. Indeed, our results are consistent with that study and another one showing that wireless audio transmitters can reduce network throughput substantially more than Bluetooth handsets [22,26].

Our results reveal several challenges that network professionals will need to resolve in order to optimize the functioning of IEEE 802.11 networks in Industry 4.0 environments. These challenges affect the four steps of site surveying, frequency planning, network testing, and troubleshooting [22]. First, sites should be surveyed before and after network deployment to ensure that specific network requirements are met. For example, some scenarios require rapid (millisecond) switching from one wireless network to another, such as when several automated guided vehicles must be coordinated as their positions change constantly [2,3]. A network hampered by the interference in our experiment would likely be unable to support such roaming. Indeed, several other industrial applications require sustained high throughput, including video transmission for tracking and critical warning systems (6–48 Mbps), as well as high-resolution thermographic videos or videos that must be uploaded from smart glasses or helmet cameras (24–48 Mbps) [5]. As our results illustrate, interference can render IEEE 802.11 networks useless for such applications, leading us to recommend that network professionals implement a quality-of-service algorithm in which, for example, they prioritize network traffic and/or distribute devices to different frequency bands [22].

Indeed, network professionals should consider running only less-important applications on the 2.4 GHz band, given that this frequency is used by wireless audio/video transmitters, audio headsets, and Bluetooth handsets. At the very least, such devices could change operating frequencies in order to avoid other wireless devices. The less-congested 5 GHz band could be reserved for critical industrial communications [22]. In addition, a possible upgrade to the latest IEEE 802.11ax standard should be considered in order to mitigate the effects of interference [38].

To facilitate network testing and troubleshooting, we recommend using a spectrum analyzer during network planning and deployment. This will help diagnose and eliminate any potential non-IEEE 802.11 radiofrequency energy on the PHY layer, which is particularly important given that this layer serves as the basis for optimal functioning of upper layers [22]. Identifying interfering devices may be easier because every device transmits a unique signal shape in the frequency spectrum. If the interference is intensive and continuous, as in the present experiments, alternative anti-jamming techniques may need to be considered [39].

Our results support a holistic approach to the design and operation of IEEE 802.11 networks, in which attention is paid not only to signal coverage but also to network performance by focusing on both the PHY and MAC layers [22]. In addition, wireless network solutions should be optimized for specific industrial applications, rather than applying a “one-size-fits-all” approach.

## 7. Conclusions

Wireless networks, including the IEEE 802.11 standards, can substantially improve the flexibility, productivity, and networking of Industry 4.0 systems. However, the challenge of interference needs to be adequately addressed in order for such networks to realize their full advantages over wired communication technologies. Our results highlight the strong, potentially debilitating effect of interference from a wireless audio transmitter on the throughput of a wireless 802.11 b/g/n network under laboratory conditions. Our results highlight the need for further research into interference and network performance, and they support a holistic approach in which networks are optimized for particular industrial applications based not only on signal coverage but also network performance. Comprehensive analysis of such interference requires examining not only the MAC layer but also the PHY layer in order to identify the specific radiofrequencies causing interference and understand how they affect network throughput. The present laboratory-based study justifies future work to test the interference of devices specific to particular Industry 4.0 contexts. Such studies may wish to measure throughput only in the local wireless network in order to eliminate the possibility of external Internet interference. Further research could also investigate the interplay between interference and other network performance metrics, such as the received signal strength indicator. Ultimately, future studies should examine the full range of interference types and specific industrial contexts, while including the latest IEEE 802.11ax standard.

## Figures and Tables

**Table 1:** Comparison of wireless communication technologies. Data from [2,8,9,10,11].

Name	Standard	Frequency Band	Theoretical Communication Range	Theoretical Data Transfer Rates
Advanced and adaptive network technology (ANT)	Proprietary (Garmin)	2.4 GHz	30 m	12.8, 20 or 60 Kbps
Bluetooth	IEEE 802.15.1	2.4/5 GHz	10 m	1, or 24 Mbps
Bluetooth low energy (BLE)	IEEE 802.15.1	2.4 GHz	50 m	125, 250, 500 Kbps; 1–2 Mbps
Long range (LoRa) WAN	LoRaWAN TS1-1.0.4	868/900 MHz	30 Km	0.3–50 Kbps
Narrowband Internet of Things (NB-IoT)	3GPP Release 13/14 (LTE Advanced Pro)	180 kHz	10 Km	26–159 Kbps
Radio-frequency identification (RFID)	ISO/IEC 24791	125 kHz/13.56 MHz/433 Hz	10 cm/1 m/20 m/100 m	5/26.48/640 Kbps
Wi-Fi	IEEE 802.11	2.4/5 GHz	10–150 m	54 Mbps to < 1 Gbps
Worldwide interoperability for microwave access (WiMAX)	IEEE 802.16	2.4/5.1–66 GHz	0.3–49 Km	1 Mbps–1 Gbps (Fixed); 50–100 Mbps (mobile)
ZigBee	IEEE 802.15.4	868/915 MHz/2.4 GHz	10–300 m	20, 40, or 250 Kbps
Z-Wave	Proprietary/ITU.G9959 PHY/MAC	900 MHz	30 m	100 Kbps

**Table 2:** General settings used for the D-Link DIR-615.

Feature	Setting
BSSID	d_8
802.11 mode	b/g/n mixed mode
Band	2.4 GHz
Channel no.	6
Channel width	22 MHz

**Table 3:** Tests of skewness or kurtosis in measurements of down- and upload throughput.

Variable	Scenario	n	Pr (Skewness)	Pr (Kurtosis)	Test Statistics	
					Adjusted chi^2^	Pr > chi^2^
Download	No interference	30	0.0265	0.5439	5.16	0.0759 *
Download	Interference	30	0.0532	0.6844	4.11	0.1283
Upload	No interference	30	0.1362	0.7623	2.56	0.2786
Upload	Interference	30	0.0626	0.3746	4.38	0.1120

Pr, probability. * *p* < 0.1.

**Table 4:** Levene’s test of the homogeneity of variance in download measurements.

Condition or Variable	Mean	Std. Dev.	Frequency
No interference	30	5.8539	30
Interference	30	1.1249	30
Total	6.3705	6.3252	60
W_0_ (based on mean)	45.6455	df (1, 58)	Pr > F = 0.0000 *
W_50_ (based on median)	14.7967	df (1, 58)	Pr > F = 0.0003 *
W_10_ (based on 10% trimmed mean)	33.0838	df (1, 58)	Pr > F = 0.0000 *

Pr, probability. * *p* < 0.001.

**Table 5:** Levene’s test of the homogeneity of variance in upload measurements.

Group	Mean	Std. Dev.	Frequency
No interference	30	3.8534	30
Interference	30	1.8239	30
Total	8.599	7.0762	60
W_0_	10.2758	df (1, 58)	Pr > F = 0.0021 *
W_50_	9.6094	df (1, 58)	Pr > F = 0.0029 *
W_10_	9.9085	df (1, 58)	Pr > F = 0.0025 *

Pr, probability. * *p* < 0.01.

**Table 6:** Comparison of download throughput between the control and interference scenarios based on a *t* test.

Condition or Variable	n	Mean	Std. Err.	Std. Dev.	[95% Conf. Interval]	
No interference	30	11.0786	1.0687	5.8539	8.8927	13.2645
Interference	30	1.6623	0.2053	1.1249	1.2422	2.0824
Combined	60	6.3705	0.8165	6.3252	4.7365	8.0044
Difference *		9.4163	1.0883		7.197	11.6356
						t = 8.6520
H_null_: diff = 0		Satterthwaite’s degrees of freedom = 31.139				
H_alternative_: diff < 0		H_alternative_: diff ! = 0			H_alternative_: diff > 0	
Pr (T < t) = 1.0000		Pr(|T| > |t|) = 0.0000 **			Pr (T > t) = 0.0000 **	

* difference (diff) = mean (control scenario)-mean (interference scenario). ** *p* < 0.001.

**Table 7:** Comparison of upload throughput between the control and interference scenarios based on a *t* test.

Group	Obs	Mean	Std. Err.	Std. Dev.	[95% Conf. Interval]	
No interference	30	14.9593	0.7035	3.8534	13.5204	16.3982
Interference	30	2.2386	0.333	1.8239	1.5576	2.9197
Combined	60	8.599	0.9135	7.0762	6.771	10.4269
Difference *		12.7206	0.7783		11.1491	14.2922
						t = 16.3426
H_null_: diff = 0		Satterthwaite’s degrees of freedom = 41.3728				
H_alternative_: diff < 0		H_alternative_: diff ! = 0			H_alternative_: diff > 0	
Pr (T < t) = 1.0000		Pr(|T| > |t|) = 0.0000 **			Pr (T > t) = 0.0000 **	

* difference (diff)= mean (Scenario 1)-mean (Scenario 2). ** *p* < 0.001.

## Data Availability

Data available upon request.
